# A Comparative Study of Post-Quantum Cryptosystems for Internet-of-Things Applications

**DOI:** 10.3390/s22020489

**Published:** 2022-01-09

**Authors:** Jose-Antonio Septien-Hernandez, Magali Arellano-Vazquez, Marco Antonio Contreras-Cruz, Juan-Pablo Ramirez-Paredes

**Affiliations:** 1 Department of Electronics Engineering, Campus Irapuato-Salamanca, University of Guanajuato, Carr. Salamanca-Valle de Santiago km 3.5 + 1.8, Comunidad de Palo Blanco, Salamanca 36885, Mexico; ja.septienhernandez@ugto.mx (J.-A.S.-H.); ma.contrerascruz@ugto.mx (M.A.C.-C.); 2 INFOTEC, Cto. Tecnopolo Sur No. 112, Aguascalientes 14050, Mexico; magali.arellano@infotec.mx

**Keywords:** post-quantum cryptography, Internet of Things, resource-constrained devices

## Abstract

The existence of quantum computers and Shor’s algorithm poses an imminent threat to classical public-key cryptosystems. These cryptosystems are currently used for the exchange of keys between servers and clients over the Internet. The Internet of Things (IoT) is the next step in the evolution of the Internet, and it involves the connection of millions of low-powered and resource-constrained devices to the network. Because quantum computers are becoming more capable, the creation of a new cryptographic standard that cannot be compromised by them is indispensable. There are several current proposals of quantum-resistant or post-quantum algorithms that are being considered for future standards. Given that the IoT is increasing in popularity, and given its resource-constrained nature, it is worth adapting those new standards to IoT devices. In this work, we study some post-quantum cryptosystems that could be suitable for IoT devices, adapting them to work with current cryptography and communication software, and conduct a performance measurement on them, obtaining guidelines for selecting the best for different applications in resource-constrained hardware. Our results show that many of these algorithms can be efficiently executed in current IoT hardware, providing adequate protection from the attacks that quantum computers will eventually be capable of.

## 1. Introduction

The Internet of Things (IoT) is the next step in the evolution of the Internet, which will create an ecosystem for connecting all sorts of devices with the objective of gathering data from the environment. This ecosystem will assist in decision-making processes, and should operate as autonomously as possible.

To achieve the goal of assisting in decision-making, the IoT has several stages [1,2,3]: sensing, which refers to the gathering and transfer of data to other platforms and devices; communication, which defines the set of technologies and ways of communication among the different devices involved; computation, which involves processing units and software that provides the computational capability to IoT devices; services, which provide all the required functionality for the IoT to work correctly; and semantics, the stage at which knowledge is extracted from data.

For a proper deployment of these components, an architecture design is required. The most common architecture for IoT comprises five layers, from the object Layer at the bottom to the business Layer at the top, as described in [1,2].

- Object layer: consists of all the objects that provide sensing, actuation, or data gathering capabilities.
- Object abstraction layer: comprises the virtual objects necessary for connecting the physical objects with the rest of the ecosystem, and provides means of interacting with them.
- Service management layer: provides a mapping system for pairing the service requester to the service providers.
- Application layer: responsible for delivering smart services to end-users, providing all the required functionality for the user’s interaction with the service and the ecosystem.
- Business layer: responsible for managing all the underlying layers, system activities, and high-level functions.

### 1.1. Basic IoT Devices and Communication Protocols

All the elements that make up the “things” part of the IoT reside at the object layer. The most basic types of IoT devices are radio frequency identifiers (RFID) [4] and wireless sensor networks (WSN) [5].

A WSN is a set of nodes capable of gathering and processing data while having wireless communication capabilities [5]. A WSN consists of three basic elements:

- A sensor field, composed of sensor nodes, for data collection. A sensor node is a low-powered, resource-constrained device with limited computational capabilities used for sensing and transmitting data. It has three main components: a processing unit, a sensing unit, and a communication unit.
- A sink or gateway for collecting and processing the data from the sensor field.
- A task manager node or server, which instructs the sink how to process the data from the sensor field and presents this information to the end-user.

At the application layer, several protocols exist for transmitting and gathering data among the whole ecosystem. The most commonly used ones are [1,2] constrained application protocol (CoAP), message queue telemetry transport (MQTT), and advanced message query protocol (AMQP). The protocol that has more acceptance in the community, MQTT, is built over TCP and is aimed at devices with weak or unreliable links using the publish/subscribe pattern for message transmission and dissemination.

Deploying all the components and devices of the IoT ecosystem will expose it to external threats, such as unauthorized parties trying to intervene and access the transmitted data, hence the importance of securing the communication between the different endpoints.

### 1.2. Quantum Computing and Shor’s Algorithm

An emerging field in computer science and physics is quantum computing. In 1985, David Deutch expanded the Church–Turing hypothesis to a physical principle [6], allowing him to propose the first universal quantum computer model, setting the foundations for making such hardware realizable.

In 1994, Peter Shor proposed an algorithm that solves the discrete-logarithm and large-integer factorization problems [7] using the capability of quantum computers for processing tasks in parallel within a single processor. Thus, such an algorithm poses a threat to cryptosystems that use such problems for guaranteeing security.

When quantum computers come to existence, they, along with Shor’s algorithm, will pose a threat to the Internet communications that use classical cryptosystems for secure key exchange, including IoT applications.

For this reason, the cryptography community has an ongoing effort to create cryptosystems that are resistant to quantum computer attacks [8]. Although there have been several proposals, there are two basic types of public-key cryptosystems based on algebraic codes and on lattices.

The primary representative for algebraic codes-based public-key cryptosystems is the McEliece cryptosystem [9], initially proposed by R. J. McEliece in 1978 as an alternative solution to the key distribution problem. The cryptosystem uses Goppa codes and the difficulty of recovering a message in the presence of *t* errors to guarantee security. As it is not based on the problem that Shor’s algorithm solves, it is considered quantum-resistant.

As for lattice-based public-key cryptosystems, their primary representative is known as the NTRU cryptosystem [10], proposed in 1998 by Hoffstein, Pipher, and Silverman. It uses polynomial algebra and reduction modulo two integers, *p*, and *q*. To provide security, the cryptosystems are based on two problems: the ability to mix polynomials independent of the modulo and the difficulty of finding extremely short vectors on a lattice. As with the McEliece cryptosystem, this also has quantum-resistance. It is worth mentioning that the hardness of finding an extremely short vector in a lattice is based on empirical observations and has no formal proof yet.

Along with community efforts to create a quantum-resistant public-key cryptosystem, and considering the time it takes to deploy a new standard, the US National Institue of Standards (NIST) began a process in 2017 [11] to create a new standard for key exchange and digital signatures that could withstand quantum computer attacks. The process is currently in its third stage, with fifteen alternatives, seven as finalists and eight as alternatives, for both key exchange and digital signatures. This standard will be deployed to the industry, and secure the communications from quantum computers attacks. This standard will also reach IoT devices and the links they use to transmit data.

For that reason, and considering that classical cryptosystems were not designed with resource-constrained devices in mind, it is worth studying the performance of the different cryptosystems on such devices.

### 1.3. Related Work

There has been little work regarding the study of the performance of the cryptosystems, especially on resource-constrained devices or methods to adapt them to it. On [12], a survey is presented on the current efforts on creating new post-quantum cryptosystems, both in the academy and the industry. From that survey, some guidelines are provided for selecting the appropriate cryptosystem but are built from the authors’ provided data.

In [13], the authors propose a method that applies a compression and error correction algorithm to ciphertexts generated by lattice-based cryptosystems, reducing the ciphertext’s size without losing information and thus diminishing the number of bytes transmitted through the network.

In [14], the authors propose an adaptation of a key management scheme known as identity-based encryption (IBE) to use, as the underlying cryptosystem, a lattice-based post-quantum one.

In [15], the authors present the basics of lattices and how cryptosystems are built from them. They also present the two problems used for the creating the ciphers, and some algorithms that use lattices for encryption. The authors then proceed to argue that lattice-based cryptosystems are more suitable for IoT devices due to their theoretical benefits: smaller key sizes, shorter encryption/decryption time, and overall less memory usage, compared to code-based cryptosystems.

### 1.4. Contributions

In this work, we begin considering the impacts on performance and energy consumption that the current post-quantum key exchange mechanisms from the NIST standardization process could have on resource-constrained devices, especially on IoT devices. For measuring the impact of the cryptosystems on IoT devices, we select first those cryptosystems most suitable for software implementations from the NIST standardization process. Afterwards, we select the relevant variables to measure, and build an IoT prototype that emulates a real-world scenario on which to test the cryptosystems. This work concludes by giving some guidelines for selecting an appropriate cryptosystem for use in IoT devices. Our contributions are summarized as follows:

- We demonstrate that post-quantum cryptosystems can be integrated in IoT devices in their current form.
- We consider algorithms that are currently participating in the NIST competition for post-quantum encryption standard, and integrate them into commonly-used IoT software and hardware.
- We measure the impact on device resource-consumption of the different post-quantum encryption algorithms.
- We provide guidelines on selecting a cryptosystem to use on the device, according to the constraints imposed on it.

### 1.5. Article Organization

This article is organized as follows. Section 2 provides some background on post-quantum cryptography and the NIST standardization process, mentioning the currently competing cryptosystems. We also introduce the prototype in use for testing, including the hardware and software required. We then explain how we obtained the data and how we performed the analysis. In Section 3, we present our results, beginning by considering some theoretical aspects and which cryptosystems are suitable to IoT devices, according to the literature. Then, we present the empirical study results and give some guidelines on which cryptosystem to select for IoT devices. Finally, in Section 4, we provide some conclusions and future work.

## 2. Methods

First, we introduce the different cryptosystems available at the NIST standardization process and briefly mention some important aspects.

Then, we introduce the system in use and its components. We introduce the hardware for the nodes, the communication protocol in use, and describe the different software components we used for building the systems and their components.

Finally, we introduce the different variables to measure and why we considered them essential, how we measured them, and how we gathered the data, explored it, and interpreted it.

### 2.1. Post-Quantum Cryptography and the NIST Standardization Process

We are interested in knowing how the cryptosystems available at the NIST standardization process will adapt to resource-constrained devices, by obtaining some first insights into whether they are suitable for such devices in their current form. From this data, optimizations could be implemented from an early stage, thus saving time and resources.

We choose to study post-quantum cryptosystems from the NIST standardization process, as some of those will be deployed to the industry and will be integrated into libraries that resource-constrained devices will use for communication over the Internet.

At the time of writing, the NIST standardization process is in its third phase, with fifteen candidates remaining, of which seven are selected as finalists and eight as possible alternatives. In Table 1, we show the finalists, and in Table 2, we show the alternatives.

As we mentioned in Section 1.2, there are two types of cryptosystems resistant to quantum computers: those based on lattices and those based on codes. For the leading contenders, we have one cryptosystem based on codes (Classic McEliece [16]) and three based on lattices (Crystal-Kyber [17], NTRU [18], and SABER [19]). For the alternative candidates, we have three based on codes (Bike [20], HQC [21], Sike [22]) and two based on lattices (FrodoKEM [23], NTRU Prime [24]).

### 2.2. Algorithms to Consider

For selecting the cryptosystems to study, we have to know first which ones are most appropriate for resource-constrained devices, and consider some of their theoretical aspects, such as strength, security level [25], and sizes of the keys and ciphertexts.

According to the literature, the cryptosystems most suitable to resource-constrained devices are those lattice-based [12]. Hence, we searched for this kind of system among the NIST competition candidates. Those based on codes are Classical McEliece [16], BIKE [20], HQC [21], and SIKE [22]; those based on lattices are CRYSTAL-KYBER [17], NTRU [18], NTRU Prime [24], SABER [19], and FrodoKEM [23]. Selecting cryptosystems that are lattice-based, and considering that smaller keys and ciphertext size are better suited for resource-constrained devices, we chose the following versions for each.

- For SABER, LightSaber with a public-key size of 672, private-key size of 992, and ciphertext of size 1312 bytes. It has security category 1.
- For CRYSTAL-KYBER, Kyber512 with a public-key size of 1632, a private-key size of 800, and ciphertext of 736 bytes. Its claimed security level is 1.
- For NTRU, NTRUhps2048509 with a public-key size of 699, a private key size of 935, and a ciphertext size of 699 bytes. It provides level 1 security.
- For NTRU Prime, NTRULPr653 with a public-key size of 897, a private-key size of 1125, and ciphertext of 1025 bytes. Its expected strength falls into category 2.
- For FrodoKEM, FrodoKEM640 with a public-key size of 9616, a private-key size of 19,888, and ciphertext size of 9720 bytes. It provides level 1 security.

They all have IND-CCA2 (indistinguishability under adaptive chosen ciphertext attack) theoretical strength. These are the selected cryptosystems we consider in our study. Next, we present the system and IoT prototype used for testing.

### 2.3. System and IoT Prototype

**Figure 1:**
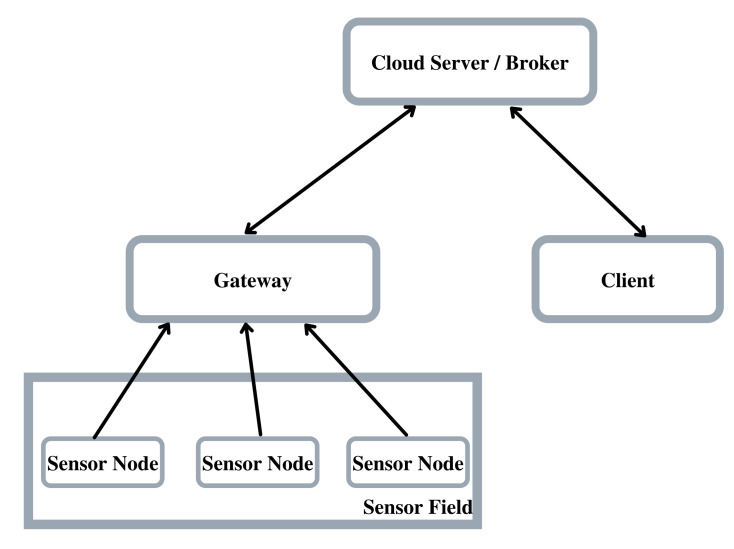
The architecture used for the tests, composed of all basic elements: a wireless sensor network, a gateway, and a cloud server.

For testing the performance of the cryptosystems, we built a small IoT system consisting of three essential components: a wireless sensor network (WSN), a gateway, and a cloud server, as shown in Figure 1. We now describe each of the components of the system.

#### 2.3.1. Hardware Setup

For the nodes that make the sensor network, we used the following hardware:

- An Arduino Nano with an ATmega328P microcontroller as the processing unit.
- The DHT22 humidity and temperature sensor, as the sensing unit.
- The RFM69HCW module, a transceiver module that implements the LoRa protocol [26], as the communication unit.

**Figure 2:**
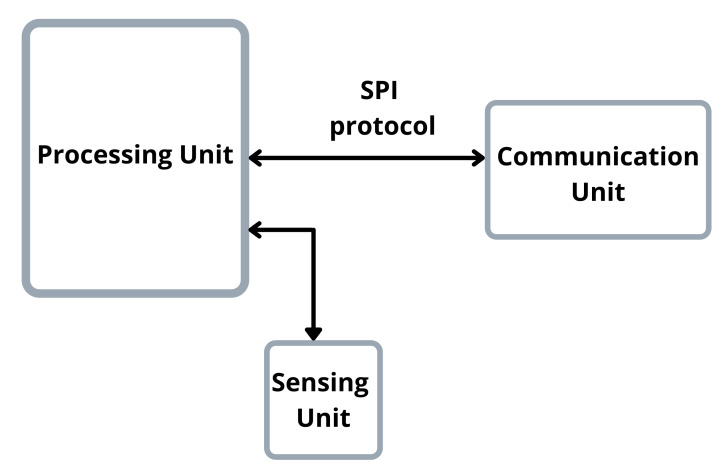
The block diagram of the sensor node. It consists of three parts, the processing unit, for which we used an Arduino Uno; the communication unit, an RFM69HCW; and a sensing unit, the DHT22 sensor.

Figure 2 shows the node’s block diagram.

**Figure 3:**
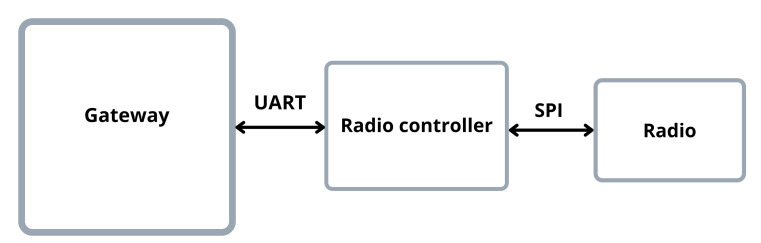
The block diagram for the gateway part of the system. No direct connection between the gateway and the radio was possible, so an intermediary device was necessary, to act as a radio controller. For the gateway, we used a Raspberry Pi 3B+; for the radio controller, we used an Arduino Uno; and an RFM69HCW for the radio.

For the gateway part of the system, we chose to use the Raspberry Pi 3B+ for its large community of users, its capability to run popular encryption libraries like OpenSSL, its Internet connection versatility, and the wide variety of communication interfaces on the board (Serial, Ethernet, USB, etc.) The Raspberry Pi is connected to an RFM modulo via an Arduino Uno, who works as a driver, as shown in Figure 3.

#### 2.3.2. Software Setup

We used several libraries to create the entire system. First, a library implementing the Transport Layer Security (TLS) 1.3 protocol; a library that implements an MQTT client; an MQTT broker; and several other libraries for use in the hardware for the sensor nodes.

For the cryptography library, we used OpenSSL version 1.1.1g, with modifications to integrate the post-quantum cryptosystems [27]. This library is used for the libraries that implement the MQTT protocol.

For the broker part of the protocol, we chose to use Mosquitto, a project by the Eclipse Foundation [28], and for the MQTT client, we used the Paho C MQTT library. For both cases, at the time when we were developing this work, neither Mosquitto nor Paho C MQTT supported the OpenSSL 1.1.1g API (which has the post-quantum algorithms integrated) and TLS 1.3 protocol, so we made the proper modifications to both of them so the experiments could be performed [29,30]. All the code used for the testing, including performance measurement, plot generation, and Arduino programming, can be found at [31]. The Mosquitto broker was set up to run on a cloud server provided by the Microsoft Azure platform.

### 2.4. Performance Measurement

One of the most limiting factors in the design process is the energy available to the device when it is resource-constrained. The energy will determine the expected life-span of the devices and will limit other requisites as well.

It has been demonstrated that, on embedded devices, the subsystems with the highest energy consumption are the CPU and the radios (such as Bluetooth and Wi-Fi) [32]. We should therefore minimize the usage of such components when designing software for resource-constrained devices. Another essential component to consider that affects the device is the memory consumption of a particular software.

To the best of the authors knowledge, there is no simple way to theoretically measure the energy consumption of the device and its different components due the complexity of modern embedded architectures. For this reason, we consider it appropriate to perform empirical tests, providing a better measurement of the energy consumed. Most of the efforts made by the community regarding energy consumption on resource-constrained devices have been on creating some framework to empirically measure the energy consumed by their components, as they execute their software [32].

We chose to study the software’s performance using the post-quantum KEMs in terms of the following components: CPU, RAM, and the network traffic. The energy consumption of such devices cannot be directly measured from software, but minimizing such a component’s usage leads to them using less energy.

To measure the different cryptosystems’ performance, we use the authors’ code provided to NIST, available on the website [33]. We also wrote software that allows us to measure the CPU and RAM consumption of the different KEMs, and used the libraries mentioned above to create a client that allows us to test the KEMs with an IoT protocol.

The program allows us to specify at compile-time the following:

- KEM to profile.
- Whether to profile RAM or CPU usage.
- If the CPU is to profile, specify the file to save the data.
- Select a platform in which to profile: x86 or ARM architectures.

It has a function for each of the KEM operations: key generation, encapsulation, and decapsulation, which allows us to profile the CPU usage separately. It executes 2000 runs for each operation (arbitrary value choice).

#### 2.4.1. Measuring Memory and CPU Usage

For measuring the program’s RAM usage, we used the command-line tool Valgrind, with the massif tool. This tool can measure both the heap and the stack portions of the memory used.

We used the system call clock\_gettime for the ARM architecture for measuring the CPU cycles and the assembly instruction rdtsc for x86 architectures. These return the number of cycles the program used. For obtaining the time in milliseconds, we used the system call gettimeofday for both architectures.

#### 2.4.2. Measuring Network Traffic

We used the companion command-line tool for Wireshark, tshark, for measuring the network usage while monitoring a connection. We then used Wireshark to obtain series of statistics regarding a connection, such as information about a conversation’s endpoints (IP and port), and data on the total number of packets, total number of bytes, number of packets transmitted from A to B and vice versa, bytes sent from A to B and vice versa, relative start (to the first connection), duration of the current connection, and bits transfer rate from A to B and vice versa.

We discarded the information on the endpoints, as it is irrelevant for the analysis. We also discarded one-way data: data from point A to point B or vice versa, as it would not give us complete information. Relative start gives us no information on the connection, either. Given that both endpoints receive the same packets in a connection, we considered only those variables that consider it whole, namely, the number of packets sent, the number of bytes, and the connection’s duration, as the more relevant parameters.

We then computed the mean, maximum, and standard deviation statistics for each variable to gain information on the average and worst-case scenario.

For comparison purposes, besides using the three selected post-quantum cryptosystems, we also used two elliptic curves available at the OpenSSL 1.1.1g implementation: P-256 and X25519, both with a key size of 256 bits and 128-bit security.

The connection for both clients (the client and the gateway on Figure 1) was initiated from the function provided by MQTT Paho C *MQTTClient_connect*. After the initial connection, we did not send any data as we were only interested in establishing a connection with the broker. The connection then includes the TCP handshake, the TLS handshake, and the connection sequence for MQTT.

### 2.5. Data Exploration and Tests Execution

To obtain information about the cryptosystems performance and to provide guidelines, we first profiled the programs on a laptop computer to obtain a general overview of the KEMs’ behavior, and then executed the tests on the proposed hardware, specifically on the gateway. This allowed us to obtain data on the performance of the cryptosystems.

We present a summary of the performance data via a set of statistics: mean, maximum, and standard deviation. We plotted the data on different graphs to visually convey information relevant to our purposes. The proposed graphs are bar graphs and line graphs.

We only present the maximum for memory, as this statistic gives us a limit on how much RAM is available to other software when the KEM is under execution. For the CPU usage, we present a summary by operation and totals.

For executing Wi-Fi tests, it is required that the mechanisms are already integrated into the OpenSSL library, as it required a fully functional implementation of the TLS protocol. On the other side, executing the tests for CPU and RAM can be performed without any external library. For those reasons, the performance tests were divided into two parts: the first part for testing the CPU and the RAM usage, and the second part for the Wi-Fi usage.

In the second part, we also compare the post-quantum mechanisms’ performance against classical ciphers. In particular, because the TLS protocol version 1.3 removed RSA for key exchange, we compared the KEMs against elliptic curves with similar security levels: the elliptic curve P-256, and the elliptic curve X25512, both with key size of 256 bits.

For both tests series, we applied these three steps several times; first, on a PC to gain a general overview of each variable’s behavior, and then on the Raspberry Pi, to obtain the real data on a device with fewer resources than an average computer. It is worth mentioning that we also considered theoretical aspects of the different cryptosystems, in particular the strength, security level in bits, and key sizes.

## 3. Results

### 3.1. Memory Usage

We now present the results from memory usage for each of the KEMs: Kyber512, NTRUhps2048509, LightSaber, NTRULPr653, and FrodoKEM640. For measuring the RAM usage, we made a single run of the program. We measured the consumption for a whole run of the key exchange process, key generation, encapsulation, and decapsulation. We report RAM usage against number of access to the memory.

**Figure 4:**
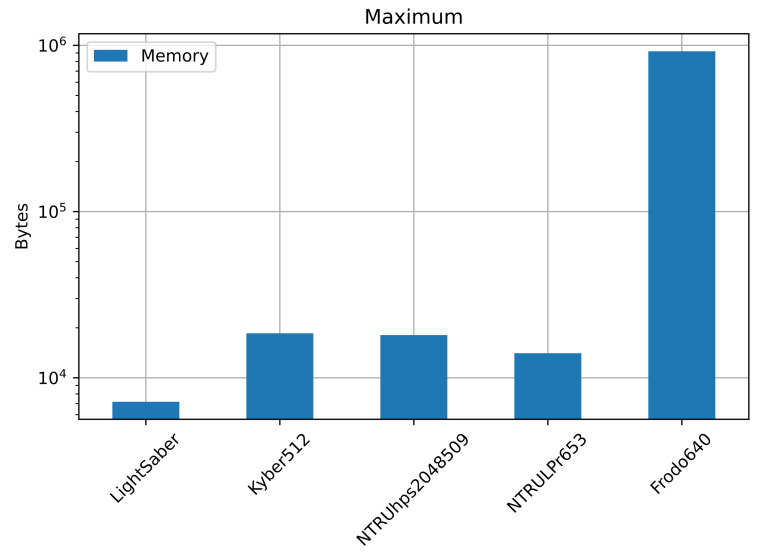
Maximum amount of memory, in bytes, that each KEM uses. We can see again that FrodoKEM640 uses the most, with close to $10^{6}$ bytes. LightSaber uses the least, with less than $10^{4}$ bytes. The other three KEMs use slightly more than $10^{4}$ bytes. The *y*-axis has logarithmic scale.

Highlighted in bold the best-performing ones, Table 3 shows the maximum amount of memory the KEM used during its execution, and we plotted it in Figure 4. LightSaber uses the least amount of memory, with a maximum of 994 bytes, followed by NTRULPr, with 14,064 bytes. The most consuming one is FrodoKEM640, with almost 1 MB of memory in use. In the middle fall NTRUhps2048509 with 18,080 bytes and Kyber512 with 18,528 bytes.

**Figure 5:**
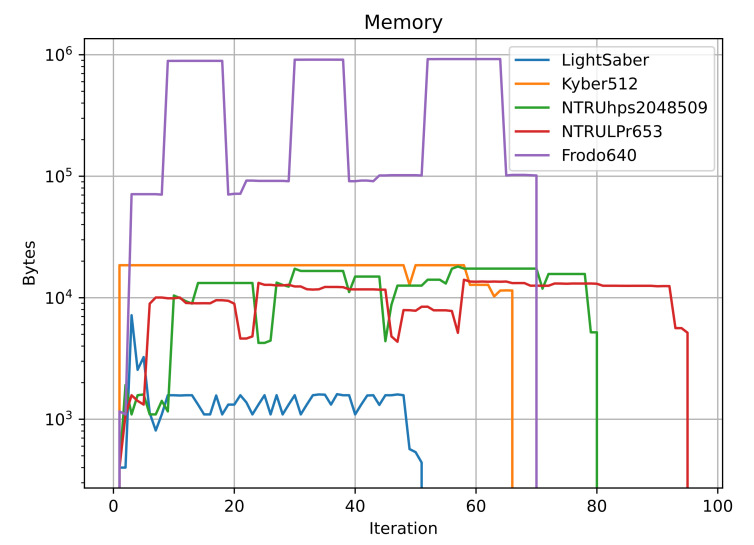
Memory usage over number of access for each KEM. The more access to memory, the more CPU cycles the program requires to access the RAM. LightSaber uses the memory for the least amount of time, while NTRULPr653 accesses it for the longest time. The other three fall in between. The *y*-axis has logarithmic scale.

Figure 5 shows how memory consumption behaves over the number of accesses to the RAM. We can see that LightSaber has the least number of accesses, followed by Kyber512, FrodoKEM640, then NTRUhps2048509, and finally NTRULPr653. Accessing memory for an extended period usually means more CPU cycles for accessing it; this can also provide some guidelines for selecting the KEM.

In terms of the number of bytes required, the best option would be LightSaber, requiring, at most, 994 bytes during execution; it uses also the RAM for the least amount of time.

### 3.2. CPU Usage

As mentioned in the Methods section, we wrote a program for measuring the CPU’s performance. We measured each operation: key generation, encryption, and decryption, with a total of 1000 runs for each one.

**Figure 6:**
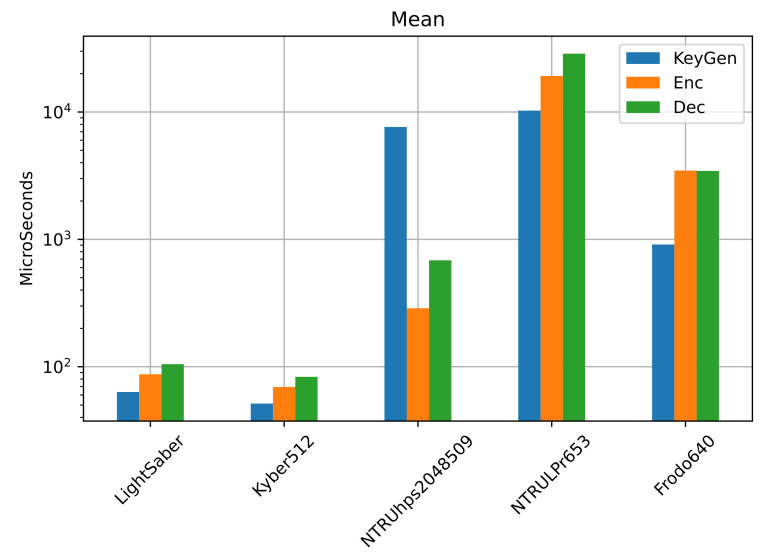
Mean usage of the CPU for each operation of each cipher. Visually, the performance of the KEMs LightSaber and Kyber51 is very similar. NTRUhps2048509 is the next in performance, followed by NTRULPr653, and then FrodoKEM640. Table 5, Table 6 and Table 7 show the standard deviation and 95% CI for *key generation*, *encryption*, and *decryption*, respectively.

Table 4 shows the total amount of CPU usage for each KEM operation, and in Figure 6, we plot the time each operation took to complete. In Table 5, Table 6 and Table 7, we show the standard deviation and the 95% CI for the operations *key generation*, *encryption*, and *decryption*, respectively. We highlighted in bold the best-performing ones.

We can see that the best-performing is Kyber512, followed by LightSaber, with a total time of 204 and 255 ms, respectively. Considering the encapsulation and decapsulation, NTRUhps2048509 performs better than NTRULPr and FrodoKEM640, but in total usage, both NTRULPr653 and FrodoKEM640 perform better than NTRUhps2048509.

In terms of execution time, Kyber512 and LightSaber are the best-performing.

### 3.3. Selection of Candidate Algorithms

For selecting a KEM to use in a resource-constrained device, we have to consider CPU and memory usage. We saw that the best-performing in CPU usage were Kyber512 and LightSaber, whereas, for the RAM, it was LightSaber and NTRUhps2048509. Our first choice here is LightSaber, as it performs well on both tests and accesses the memory for the least time. We have our first KEM to discard, FrodoKEM640, as it was the worst-performing for both tests.

We still have Kyber512, NTRUhps2048509, and NTRULPr653. Kyber512 performed the best in terms of CPU usage, but it was fourth in memory; NTRULPr653 performed third in memory usage but was the worst in CPU. NTRUhps2048509 was the second-best-performing for the RAM and the fourth in memory, and has the advantage of being a finalist and possibly reaching the industry earlier. For that reason, and with NTRULPr653 being the worst-performing in terms of CPU usage, we chose NTRUhps2048509 as an option, and discarded NTRULPr653. For the case in which the CPU usage is vital, we can choose Kyber512, as it uses the least amount, and its RAM usage is one of the best, just above LightSaber.

NTRULPr653 could be a good option, given that its performance in CPU usage is better. Nonetheless, we only selected Kyber512, LightSaber, and NTRUhps2048509 for use on resource-constrained devices. In the following, we present the results of using the KEMs on an IoT device.

### 3.4. Network Traffic Statistics

We now present the results regarding the data related to the connection. We begin by presenting the results on the number of packets, we then present the packet size results, and finally the duration of the connection.

#### 3.4.1. Number of Packets

**Figure 7:**
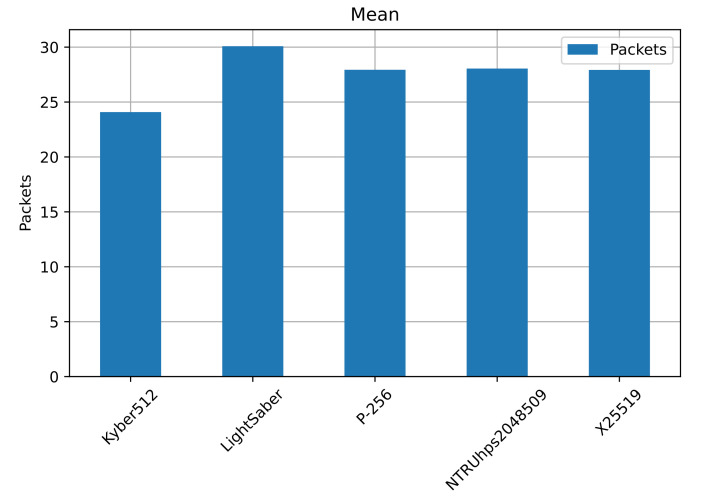
The maximum number of packets transmitted during the connection. LightSaber required the maximum number of packets to transmit during the connection, while Kyber512 used the least. Table 8 shows the 95% CI of the number of packets transmitted.

Table 8 shows the statistics for the number of packets sent over the connection, with the best-performing ones highlighted in bold, all computed from a benchmark of 1000 runs, and Figure 7 shows the mean value for the number of packets transmitted.

Kyber512 has the least amount of packets sent, with a mean of 24 packets per connection and a maximum of 27. With a value of 0.2326, the standard deviation tells us that the number of packets would not exceed the given values for the mean and maximum. It is followed by the elliptic curves P-256 and X25519, with a mean of 28 packets and a maximum of 30. The curve P-256 has a standard deviation of 0.36, and the curve X25519 of 0.41, which, added to the mean and maximum value, would not surpass the given values by much.

Then comes the NTRUhps2048509 with a mean of 28 and a maximum of 32, with a standard deviation of 0.35. This last value tells us that the connection might require one more packet. LightSaber performs the worst in terms of the number of packets, having a mean of 30, maximum of 36, and standard deviation of 0.58.

We can see that the best-performing in the number of packets sent is Kyber512, followed by the elliptic curves, NTRU and LightSaber. We consider the packet size next.

#### 3.4.2. Packet Size

**Figure 8:**
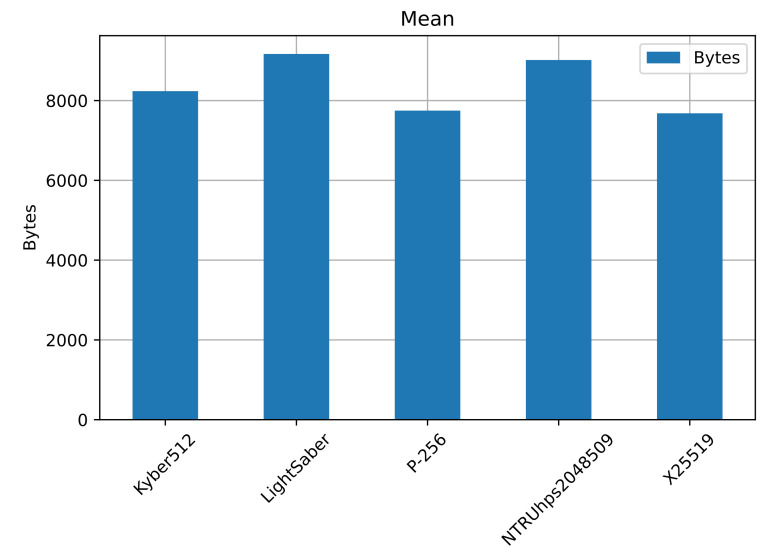
Number of packets transmitted during the connection. The elliptic curves performed better than the post-quantum KEMs. Kyber512 still performs better, followed by NTRUhps2048509 and LightSaber. In Table 9, we show the 95% CI for the packet size.

Table 9 shows the corresponding statistics for the packet size on the connection, highlighting the best-performing ones in bold. Figure 8 shows the mean number of bytes for all the connections, for a total of 1000 runs.

The elliptic curves outperformed the post-quantum mechanisms; still, Kyber512 outperformed both NTRUhps2048509 and LightSaber, with a mean of 8263, a maximum of 8442, and a standard deviation of 31.25 packets. Then comes NTRUhps2048509, followed by LightSaber. The difference between both is not large, but NTRUhps2048509 still outperforms LightSaber. We now consider the last variable for the Wi-Fi usage, the connection duration.

#### 3.4.3. Connection Duration

**Figure 9:**
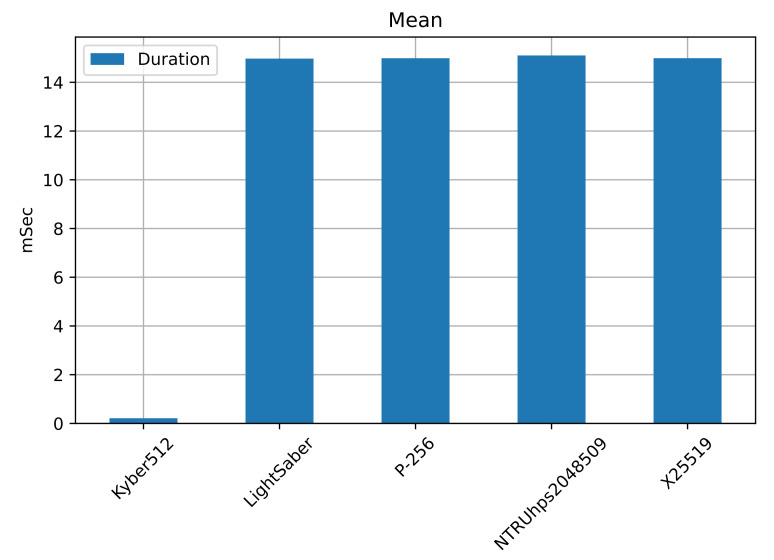
The mean value of the connection is almost the same for all the KEMS, with an average of a little more, of 14 ms. The minimum value is achieved by Kyber512, with less than 2 ms. In Table 10, we show the 95% CI for the connection duration.

Highlighting in bold the best-performing ones, Table 10 shows the statistics on the duration of the connection for a total of 1000 runs. Figure 9 shows the average mean value for each KEM used. The unit of time is milliseconds.

We can see that, except for Kyber512, all KEMs have a duration of about 14 ms on this hardware, with a similar maximum value. The best-performing is Kyber512, with a mean of 0.21 ms and a maximum of 2 ms, considerably outperforming the other KEMs. Even considering the standard deviation, the duration still outperforms the rest by a significant factor.

Again, Kyber512 outperformed NTRUhps2048509 and LightSaber, and, as with the number of packets, it outperformed the elliptic curves. The post-quantum KEMs have the following order in performance: the best being Kyber512, then NTRUhps2048509, and finally LightSaber.

Regarding the connection data, we can see that the post-quantum KEMs have the following order for all cases: Kyber512, NTRUhps2048509, and LightSaber. In comparison, the elliptic curves outperform the post-quantum KEM only in the number of packets but are outperformed by Kyber512 in the other two cases, and outperform NTRUhps2048509 and LightSaber.

### 3.5. Guidelines for Selecting a KEM

Given that we have presented some theoretical aspects and the empirical performance of the different KEMs, we now introduce some guidelines for selecting an appropriate KEM for use in resource-constrained devices.

Table 11 shows a summary of some theoretical aspects of the cryptosystems; we present their strength, security level, keys, and ciphertext size. Table 12 and Table 13 show a summary of the KEM’s performance. Considering the results, we present some guidelines for selecting a KEM in Table 14. We provide them only for those that we tested for CPU, RAM, and network usage. Let us recall that NTRU Prime is an excellent option to consider, as it has good performance and the most significant security level.

When minimal energy is required, we recommend to consider Kyber512, as it uses the least CPU and network capacity, the components that require the most energy. For minimal memory usage, we select LightSaber; it should be used when energy can be traded with performance, as this KEM uses the most the CPU. NTRUhps2048509 is the worst-performing overall and should be considered only when the previous options are not available.

## 4. Conclusions

Once quantum computers come to existence, they will threaten all communications over the Internet, including IoT devices. For that reason, we considered the impact that post-quantum cryptosystems might have on resource-constrained and Internet of Things devices. We chose to study the mechanisms available at the US National Institute of Standards standardization process because those will be deployed to the industry and used by all users connected to the Internet.

From all the mechanisms available at the process, we chose to study the lattice-based ones, as those are more suitable for resource-constrained devices, according to the literature. From those, we chose the versions with the lowest security level that can still be considered safe for everyday use, as those have a smaller key size, translating into less computational resources required.

For studying the impact of the mechanisms on devices with low resources, we considered the performance from different aspects: RAM, CPU, and network usage. We considered these three variables to be the most significant, as the last two impact the devices’ energy consumption, and the RAM determines the minimal requirements that the device should have to run the corresponding mechanism.

When studying the KEMs’ performance for network usage, we compared them against elliptic curves implemented in the OpenSSL cryptography library. We see that, in general, the elliptic curves performed similarly to the post-quantum KEMs, for implementations not necessarily optimized for devices with low resources. These results indicate the need for more optimizations, so devices with lower resources can utilize them without sacrificing much energy and resources.

We focused solely on resource-constrained devices, as those are typically used for the Internet of Things. However, the performance study can be extended to include all the currently competing KEMs, including the versions with higher security settings and the code-based KEMs. For the systems under consideration for this study, we found that the best-performing KEMs are Kyber512 and LightSaber, although there is a tradeoff between energy consumption and memory storage.

It is also worth studying how feasible the implementation is of such KEMs on hardware, how they impact the performance of the device, and how they impact energy consumption. We considered that it is also worth trying to implement one of the KEMs with the higher security settings while maintaining minimal energy consumption and not surpassing the energy required by the software implementations with the lowest settings.

## Figures and Tables

**Table 1:** The current finalists of the NIST standardization process.

Public-Key Encryption/KEM	Digital Signatures
Classic McEliece	CRYSTALS-DILITHIUM
CRYSTALS-KYBER	FALCON
NTRU	Rainbow
Saber	

**Table 2:** The currently alternate candidates of the NIST standardization process.

Public-Key Encryption/KEM	Digital Signatures
BIKE	GeMSS
FrodoKEM	Picnic
HQC	SPHINCS+
NTRU Prime	
SIKE	

**Table 3:** The maximum amount of memory each KEM uses. FrodoKEM640 uses the most memory, whilst the KEM that uses the least is LightSaber. The other three use approximately the same amount of memory.

KEM	Maximum Amount of Memory (bytes)
**LightSaber**	**994**
Kyber512	18,528
NTRUhps2048509	18,080
**NTRULPr653**	**14,064**
FrodoKEM-640	921,360

**Table 4:** The mean value of CPU usage, measuring the total number of milliseconds required to complete each of the operations. The standard deviation and 95% confidence interval (CI) for each of the operations can be seen in Table 5, Table 6 and Table 7.

KEM	Key Generation	Encryption	Decryption	Total
LightSaber	63.3441	87.2609	104.7546	255.3597
**Kyber512**	**51.3695**	**69.3704**	**83.3001**	**204.0401**
NTRUhps2048509	7626.0645	288.0449	684.8025	8598.9121
NTRULPr653	10,251.6578	19,191.6461	28,705.7693	58,149.0733
FrodoKEM640	911.0205	3468.2189	3444.5259	7823.7653

**Table 5:** The standard deviation and 95% CI values of CPU usage for the operation *key generation*, for each of the KEMs.

KEM	Standard Deviation	95% CI
LightSaber	10.1671	(60.5784, 61.8387)
**Kyber512**	**7.9088**	**(49.5813, 50.56172)**
NTRUhps2048509	280.9294	(7601.0573, 7635.8811)
NTRULPr653	717.2908	(9921.4756, 10,010.3903)
FrodoKEM640	28.4814	(907.0962, 910.6267)

**Table 6:** The standard deviation and 95% CI values of CPU usage for the operation *encryption*, for each of the KEMs.

KEM	Standard Deviation	95% CI
LightSaber	13.2624	(83.5572, 85.2012)
**Kyber512**	**10.1743**	**(67.0337, 68.2949)**
NTRUhps2048509	15.4254	(286.9363, 288.8485)
NTRULPr653	1221.7108	(18,604.4936, 18,755.9356)
FrodoKEM640	57.8281	(3461.9001, 3469.0684)

**Table 7:** The standard deviation and 95% CI values of CPU usage for the operation *decryption*, for each of the KEMs.

KEM	Standard Deviation	95% CI
LightSaber	15.6096	(100.2681, 102.2031)
**Kyber512**	**11.4246**	**(80.6816, 82.0978)**
NTRUhps2048509	31.0725	(682.0771, 685.9288)
NTRULPr653	1651.7193	(27,878.7323, 28,083.4778)
FrodoKEM640	54.3391	(3438.7026, 3445.4384)

**Table 8:** Number of packets transmitted during the connection. The best-performing is Kyber512, followed by the elliptic curves. The worst-performing is LightSaber, followed by NTRUhps2048509.

KEM	Mean	Maximum	Standard Deviation	95% CI
**Kyber512**	**24.086**	**27**	**0.2326**	**(24.0716, 24.1004)**
LightSaber	30.086	36	0.5818	(30.0499, 30.1221)
**P-256**	**27.938**	**30**	**0.3579**	**(27.9158, 27.9602)**
NTRUhps2048509	28.051	32	0.9249	(27.9939, 28.1083)
X25519	27.926	30	0.4129	(27.9004, 27.9516)

**Table 9:** The mean and maximum number of bytes each KEM sends during the connection. The elliptic curves perform better then the post-quantum ones. Kyber512 performs best, followed by NTRUhps2048509 and LightSaber.

KEM	Mean	Maximum	Standard Deviation	95% CI
**Kyber512**	**8236.481**	**8442**	**31.2484**	**(8234.4812, 8238.3547)**
LightSaber	9168.695	9556	38.4691	(9166.3132, 9171.0768)
**P-256**	**7748.311**	**7876**	**23.5715**	**(7746.85, 7749.7719**
NTRUhps2048509	9016.969	9284	278.1027	(8999.7323, 9034.2057)
X25519	7681.331	7807	627.1832	(7642.4385, 7720.1835)

**Table 10:** Statistics on the connection durations, for a better appreciation of its behavior. The best performing is Kyber512, followed by the elliptic curves. The worst is NTRUhps2048509, followed by LightSaber.

KEM	Mean	Maximum	Standard Deviation	95% CI
**Kyber512**	**0.2133**	**2.8836**	**0.0931**	**(0.2075, 0.2191)**
LightSaber	14.9727	15.8695	0.0353	(14.9705, 14.9748)
**P-256**	**14.9884**	**15.1039**	**0.0147**	**(14.9875, 14.9893)**
NTRUhps2048509	15.1022	146.4901	4.1642	(14.8441, 15.3603)
X25512	14.9901	16.2665	0.0455	(14.9873, 14.9929)

**Table 11:** Summary of the theoretical aspects of the key exchange mechanisms considered so far, including key size and security level in bits. Note that all mechanisms have IND-CCA2 theoretical strength.

KEM	Security Level (bits)	Key Size (Public/Private)
Kyber512	128	800/1623
LightSaber	128	672/992
NTRUhps2048509	128	699/935
NTRULPr652	192	897/1125
FrodoKEM640	128	9616/19,888
P-256	128	256
X25519	128	256

**Table 12:** Summary of the memory and CPU usage for each of the KEMs involved. For the CPU usage, we present the total amount of time the KEMs use it.

KEM	Memory (bytes)	CPU (ms)
Kyber512	18,528	204.0401
LightSaber	994	255.3597
NTRUhps2048509	18,080	8598.9120
NTRULPr653	14,064	58,149.0733
FrodoKEM640	921,360	7823.7653

**Table 13:** Summary of the bytes, packets and the duration of the connection for each test. We present the mean value for each field.

KEM	Bytes	Packets	Duration (ms)
Kyber512	8236	24	0.2133
NTRUhps2048509	9016	28	15.1022
P-256	7748	28	14.9884
X25519	7681	28	14.9901

**Table 14:** General guidelines for selecting the appropiate post-quantum cryptosystem for resource-constrained devices, according to its performance and security.

KEM	Advantages	Disadvantages	Guidelines
Kyber512	Minimal usage of CPU	Uses the most memory	Use for minimal energy requirement
	Minimal usage of Wi-Fi	Greater public-key size	Use for fast computing and handshake
	Smallest private key		Use when suficient memory available
	Fastest connection		
LightSaber	Minimal usage of the memory	Greater Wi-Fi usage	Use when little memory is available
	Smaller public-key size	Greater CPU usage	Use when there is sufficient energy available
			Use when energy requirements can be traded
			off with resource requirements
NTRUhps2048509	Smaller private-key size	Worst-performing overall	Use when no other available
	Use less memory than Kyber512		

## Abbreviations

The following abbreviations are used in this manuscript:

KEM	Key exchange mechanism
IoT	Internet of Things
MQTT	Message queue telemetry transport
