# Correction: Septien-Hernandez et al. A Comparative Study of Post-Quantum Cryptosystems for Internet-of-Things Applications. *Sensors* 2022, *22*, 489

**DOI:** 10.3390/s25113326

**Published:** 2025-05-26

**Authors:** Jose-Antonio Septien-Hernandez, Magali Arellano-Vazquez, Marco Antonio Contreras-Cruz, Juan-Pablo Ramirez-Paredes

**Affiliations:** 1 Department of Electronics Engineering, Campus Irapuato-Salamanca, University of Guanajuato, Carr. Salamanca-Valle de Santiago km 3.5 + 1.8, Comunidad de Palo Blanco, Salamanca 36885, Mexico; ja.septienhernandez@ugto.mx (J.-A.S.-H.); ma.contrerascruz@ugto.mx (M.A.C.-C.); 2 INFOTEC, Cto. Tecnopolo Sur No. 112, Aguascalientes 14050, Mexico; magali.arellano@infotec.mx

## Error in Table

In the original publication [1], there was a mistake in Table 11. The public and private key sizes for the Kyber512 KEM appeared swapped. The corrected Table 11 appears below.

The authors state that the scientific conclusions are unaffected. This correction was approved by the Academic Editor. The original publication has also been updated.

## Figures and Tables

**Table 11:** Summary of the theoretical aspects of the key exchange mechanisms considered so far, including key size and security level in bits. Note that all mechanisms have IND-CCA2 theoretical strength.

KEM	Security Level (bits)	Key Size (Public/Private)
Kyber512	128	800/1623
LightSaber	128	672/992
NTRUhps2048509	128	699/935
NTRULPr652	192	897/1125
FrodoKEM640	128	9616/19,888
P-256	128	256
X25519	128	256

## References

[1] Septien-Hernandez J.-A., Arellano-Vazquez M., Contreras-Cruz M.A., Ramirez-Paredes J.-P. (2022). A Comparative Study of Post-Quantum Cryptosystems for Internet-of-Things Applications. Sensors.

